# *Streptococcus agalactiae *in adults at chiang mai university hospital: a retrospective study

**DOI:** 10.1186/1471-2334-11-149

**Published:** 2011-05-25

**Authors:** Romanee Chaiwarith, Waree Jullaket, Manasanant Bunchoo, Nontakan Nuntachit, Thira Sirisanthana, Khuanchai Supparatpinyo

**Affiliations:** 1 Department of Medicine, Faculty of Medicine, Chiang Mai University, Chiang Mai, Thailand

## Abstract

**Background:**

Infection caused by *Streptococcus agalactiae*, a Group B streptococcus, is an emerging disease in non-pregnant adults. This study describes the epidemiological, clinical, and microbiological characteristics of *S. agalactiae *infection in adult patients in northern Thailand.

**Methods:**

A retrospective study was conducted between January 1, 2006 and December 31, 2009 at Chiang Mai University Hospital among patients aged ≥15 years, whose clinical specimens obtained from normally sterile sites grew *S. agalactiae*.

**Results:**

One-hundred and eighty-six patients and 197 specimens were identified during the 4-year period. Among 186 patients, 82 were documented as having invasive infection; 42 patients were male (51.2%) with the mean age of 48.5 ± 19.4 years (range 17, 83). Fifty-three patients (64.6%) had underlying medical conditions; 17 patients (20.7%), 10 (12.2%), 8 (9.7%) had diabetes, chronic renal diseases, and malignancy, respectively. Among 40 patients (48.8%) with bloodstream infection, no other site of infection was determined in 29 (35.4%) patients. In the remaining 11 patients, 5 patients (6.1%), 5 (6.1%), and 1 (1.2%) had meningitis, arthritis, and meningitis with arthritis, respectively. Forty-two patients (51.2%) presented with localized infection, i.e., subcutaneous abscess (19 patients, 23.2%), chorioamnionitis (10 patients, 12.2%), urinary tract infection (5 patients, 6.1%), arthritis (3 patients, 3.7%), meningitis (2 patients, 2.4%), and spontaneous bacterial peritonitis, uveitis, and tracheobronchitis (1 patient each, 1.2%). The overall mortality was 14.6% (12 patients).

**Conclusions:**

*S. agalactiae *infection is a growing problem in non-pregnant patients, particularly in those with underlying medical conditions. Physicians should add *S. agalactiae *infection in the list of differential diagnoses in patients with meningitis and/or septicemia.

## Background

*Streptococcus agalactiae*, a group B, β-hemolytic streptococcus, is a well-known cause of postpartum infection and neonatal sepsis[1,2]. It colonizes in the gastrointestinal and urinary tract in healthy adults as well as the genital tract in healthy women[1,2]. Recently, the number of cases of invasive infection caused by *S. agalactiae *in non-pregnant adults is increasing; [2-10] the majority of patients had underlying medical conditions including diabetes, malignancy, genitourinary abnormalities, neurologic deficits, cirrhosis, renal dysfunction, steroid uses, and AIDS[2,3,6-8,11]. Clinical manifestations of invasive *S. agalactiae *infection vary widely depending on the sites of infection. Primary bacteremia without any obvious source is a common presentation in non-pregnant adults[2,3,6,7,10]. Endocarditis and meningitis have also been observed[3,6-9]. A myriad of virulence factors are crucial for its ability to cause invasive disease; these include but are not limited to the pore-forming toxins and the sialic acid-rich capsular polysaccharide[12,13]. The mortality rate ranges from 3-47% and is highest in elderly patients with underlying medical conditions[2,3,5,6,8-10]. This study aimed to describe the epidemiological, clinical, and microbiological data of invasive *S. agalactiae *infection in adult patients admitted to Chiang Mai University Hospital, a tertiary care center in Northern Thailand.

## Methods

### Study design and population

A retrospective study was conducted among patients aged ≥15 years whose clinical specimens obtained from sterile sites grew *S. agalactiae*. The study was conducted between January 1, 2006 and December 31, 2009 at Chiang Mai University Hospital, an 1800-bed, tertiary-care hospital in Northern Thailand. Clinical data were retrospectively collected using a preprinted data collection form.

### Definitions

**Colonization **was defined as the isolation of microorganisms from clinical specimens other than blood without clinical signs or symptoms[14].

**Infection **was defined as the isolation of microorganisms from sterile sites accompanying clinical signs and symptoms[14].

**Specimens from sterile sites **included blood, cerebrospinal fluid (CSF), and body fluid or pus taken from normally sterile sites such as joint, chorioamniotic fluid, urine, and peritoneal fluid.

### Microbiological methods

The blood agar subcultures from clinical specimen after 24-hour incubation at 35 to 37°C in 5% CO_2 _atmosphere were determined for group B streptococci (*S. agalactiae*). Agar plates with visible microorganism growth were observed for large, gray, translucent colonies with a narrow or no zone of beta-hemolysis, gram-positive cocci in pairs and chains. The biochemical method was used to identify the species of group B streptococci[15-17].

Antimicrobial susceptibility test was performed by agar disk diffusion method according to the Clinical and Laboratory Standards Institute (CLSI)[18]. Susceptibility testing for penicillin, ampicillin, levofloxacin, tetracycline, and vancomycin was routinely performed; susceptibility to ceftriaxone and cefotaxime was done upon request by attending physicians. All the identification and susceptibility procedures were performed at the central diagnostic laboratory, Chiang Mai University hospital. These methods were not changed during the study period.

The study was approved by the Faculty of Medicine, Chiang Mai University Ethical Committee.

### Statistical analysis

Clinical data were presented in numbers (%); mean and standard deviation (SD); and median and interquartile range (IQR) as appropriate. Comparisons of demographic data and clinical characteristics between groups of patients who survived vs. those who died were performed using Student's t-test, Mann-Whitney U test, Chi-square test or Fisher's exact test as appropriate. Univariate analysis was performed to determine predicting factors for fatal outcome. Variables with p-value < 0.10 from the univariable analysis were then tested in multivariable models. A two-sided test at a p-value of < 0.05 was used to indicate statistical significance. All statistical analyses were performed using Stata statistical software version 10.0 (Stata Statistical Software: Release 10.0, Stata Corporation, College Station, TX, 2007).

## Results

### Demographic data

**Figure 1:**
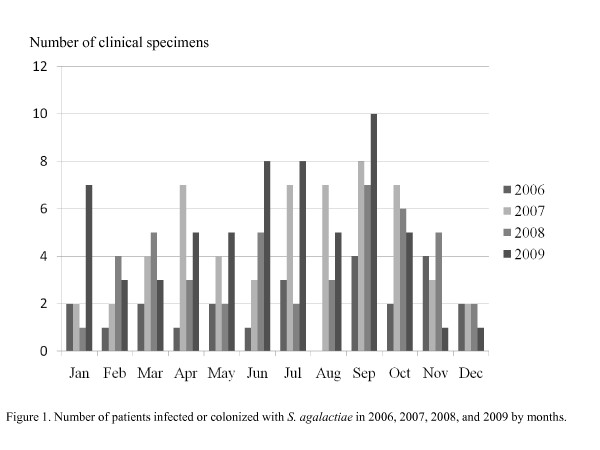
**Number of patients infected or colonized with *S. agalactiae *in 2006, 2007, 2008, and 2009 by month**.

One hundred and eighty-six patients and 197 specimens were identified during the 4-year period; the numbers of cases each year were 24, 56, 45, and 61, respectively. There were considerably more cases in September as shown in Figure 1. Among the 186 patients, 82 were determined as having invasive *S. agalactiae *infection whereas 104 patients having *S. agalactiae *colonization.

From the total of 186 patients, 84 were males (45.2%) and the median age was 52 years (IQR 41, 66). One hundred and twenty-three patients (66.1%) had underlying diseases including diabetes (38 patients, 20.4%), non-hematologic malignancy (27 patients, 14.5%), hematologic malignancy (8 patients, 4.3%), chronic kidney diseases (21 patients, 9.1%), cirrhosis (2 patients, 1.1%), thalassemia (2 patients, 1.1%), and neurogenic bladder (1 patient, 0.5%). There were 12 pregnant women (6.5%); 4 patients (2.2%), 1 (1.1%), and 7 (3.8%) were in the first, second, and third trimester, respectively.

The types of clinical isolates were urine (79 patients), pus from sterile sites (41), blood (40), chorioamniotic fluid (10), sputum (9), CSF (8), joint fluid (8), peritoneal fluid (1), and vitreous (1). Isolation of *S. agalactiae *from multiple sites was observed in some patients, i.e., from blood and CSF (5 patients), from blood and joint fluid (5 patients), and from blood, CSF, and joint fluid (1 patient).

Among the 82 patients with invasive *S. agalactiae *infection, the numbers of cases were 6, 23, 20, and 33 in 2006, 2007, 2008, and 2009, respectively. Forty-two patients (51.2%) were male and the median age was 50 years (IQR 29, 63).

### Clinical data

Among 82 patients who had invasive *S. agalactiae *infection, 40 patients (48.8%) presented with bloodstream infection. No other site of infection was determined in 29 (35.4%) of these 40 patients. In the remaining 11 patients, 5 patients (6.1%), 5 (6.1%), and 1 (1.2%) had meningitis, arthritis, and meningitis with arthritis, respectively. Forty-two patients (51.2%) presented with localized infection, i.e., subcutaneous abscess (19 patients, 23.2%), chorioamnionitis (10 patients, 12.2%), urinary tract infection (5 patients, 6.1%), arthritis (3 patients, 3.7%), meningitis (2 patients, 2.4%), and spontaneous bacterial peritonitis, uveitis, and tracheobronchitis (1 patient each, 1.2%).

Of the 8 patients who had meningitis, all had fever, 5 had headache, and 4 had altered mentation. The mean open pressure and close pressure when lumbar puncture was performed were 27.8 ± 6.4 and 22.8 ± 6.1 cm, respectively. The mean CSF protein was 538.1 ± 419.9 mg/dL and the mean CSF glucose was 26.1 ± 24.2 mg/dL.

Of the 8 patients who had arthritis, 6 had fever. All patients had monoarthritis including knee (2 patients), ankle (2 patients), hip (2 patients), shoulder (1 patient), and distal interphalangeal joint (1 patient). Joint fluid analysis was not done in 5 patients. The white blood cell counts in joint fluid from 3 patients were 17,800 (neutrophil 83%), 30,800 (neutrophil 100%), and 98,000 cells/mm^3 ^(neutrophil 96%).

All 5 patients with urinary tract infection had fever and pyuria. The white blood cells in urine were 50-100 cells/high power field (HPF). Chorioamnionitis was diagnosed in 10 pregnant women; 2 were in first trimester, 1 was in second trimester, and 7 were in third trimester of pregnancy. Six patients presented with premature rupture of membranes, 2 patients had abdominal pain after non-medical abortion, and 1 patient presented with fetal death in utero.

The demographic data and clinical characteristics were shown in Table 1.

**Table 1:** Demographic data and patient characteristics

Characteristics	Urinary tract infection (N = 5)	Bloodstream Infection (N = 29)	Meningitis^1 ^ (N = 8)	Arthritis^2 ^ (N = 8)	Chorio- amnionitis (N = 10)	Subcutaneous Abscess (N = 19)
Male: female	3:2	16:13	7:1	3:5	0:12	1.2:1
Age (years) (median, IQR)	62 (57, 71)	51 (30, 68)	55.5 (31, 64)	52 (44, 62)	28 (24, 29)	53 (45, 63)
Signs/symptoms (N, %)						
Fever (Body temperature ≥ 38°C)	5	22 (75.9)	8 (100.0)	6 (75.0)	5 (41.7)	14 (73.7)
Headache	0	3 (10.3)	5 (62.5)	0 (0)	0	0
Arthritis	0	0 (0)	0 (0)	8 (100)	0	0 (0)
Alteration of consciousness	0	2 (6.9)	4 (50)	0	0	0
Laboratory findings (mean ± SD or median, IQR)						
Hemoglobin (g/dL)	9.8 ± 2.4	12.6 ± 2.7	12.9 ± 2.3	11.6 ± 2.8	12.1 ± 1.6	11.9 ± 1.6
White blood cell count	7700	9800	10600	13900	13960	15500
(cells/mm^3^)	(6970, 9340)	(8100, 14300)	(8800, 11550)	(12600, 17180)	(10980, 14600)	(12000, 23300)
Serum creatinine (mg/dL)	2.1 (1.0, 2.5)	1.1 (0.9, 1.4)	1.0 (0.9, 1.2)	1.4 (0.9, 2,2)	-	1.4 (1.0, 1.9)
Death (N, %)	1 (20)	5 (17.2)	3 (37.5)	2 (25)	0 (0)	1 (5.3)

^1 ^Included 5 patients who had bloodstream infection and meningitis, 1 patient who had bloodstream infection, meningitis, and arthritis, and 2 patients who had isolated meningitis

^2 ^Included 5 patients who had bloodstream infection and arthritis and 3 patients who had isolated arthritis

All clinical isolates were sensitive to penicillin, ampicillin, levofloxacin, and vancomycin. All isolates were resistant to tetracycline. All patients who had infection received antimicrobial therapy; 40, 12, 6, and 6 patients received ceftriaxone, ampicillin, vancomycin, and clindamycin, respectively (Table 2).

**Table 2:** Medical treatment of 82 patients infected with *S. agalactiae*

Antibiotics	Number of patients (%)
**β-lactams**	
Penicillim G sodium	9 (11.0)
Ampicilin	12 (14.6)
Amoxicillin/clavulanic acid	1 (1.2)
Cloxacilin	3 (3.7)
Cefazolin	1 (1.2)
Ceftriaxone	40 (48.8)
Piperacillin/tazobactam	1 (1.2)
Meropenem	1 (1.2)
Imipenem	1 (1.2)
**Other antimicrobial groups**	
Clindamycin	6 (7.3)
Vancomycin	6 (7.3)

### Predicting factors of fatal outcome

Seventy of 82 patients (85.4%) fully recovered from invasive *S. agalactiae *infection. The overall mortality was 14.6% (12 patients). The patients who died had bloodstream infection (n = 5), bloodstream infection with meningitis (n = 3), arthritis (n = 2), urinary tract infection (n = 1), and subcutaneous infection (n = 1), respectively. The demographic data and clinical characteristics of those 12 patients who died were shown in Table 3.

**Table 3:** Characteristics of 12 death patients

No.	Sex	Age (years)	Site of infection	Underlying diseases	Signs/symptoms	Laboratory findings				Length of hospital stay (days)
							
						White blood cells (cells/mm^3^)	Platelets (cells/mm^3^)	PTT^1^ (seconds)	Creatinine (mg/dL)	
1	M	47	Bloodstream	No	Myalgia	8,400	97,000	34.1	3.2	1
2	M	47	Bloodstream	No	Myalgia, arthralgia	23,900	38,000	45.4	1.9	4
3	M	56	Bloodstream	Chronic alcohol drinking	Myalgia, arthralgia	7,900	16,000	51.5	2.5	1
4	M	66	Bloodstream	Chronic alcohol drinking	Fever, arthralgia, myalgia	3,800	47,000	92.3	1.2	1
5	M	68	Bloodstream	No	Fever	25,000	15,000	50.5	1.5	1
6	M	66	Bloodstream and meningitis	CVA	Fever, headache, stiffness of neck	11,900	103,000	31.4	1.0	49
7	M	39	Bloodstream and meningitis	HIV	Fever, headache, stiffness of neck, altered mentation	7,800	65,000	41.4	0.9	3
8	M	62	Bloodstream and meningitis	Hematologic malignancy	Fever, headache	12,700	25,000	-	1.1	48
9	F	50	Arthritis	Chronic renal failure, cancer	Arthritis	17,800	567,000	21.2	4.2	17
10	F	56	Arthritis	No	Arthritis	17,800	207,000	-	0.7	1
11	F	63	Urinary tract	Renal stone	Fever, dyspnea	9,340	14,000	53.4	2.1	150
12^2^	M	59	Subcutaneous abscess	Diabetes	Fever, subcutaneous abscess	14,300	127,000	26.2	5.3	3

Abbreviation: PTT, partial thromboplastin time; CVA, cerebrovascular accident; HIV, human immunodeficiency virus

^1 ^control range 24-34 seconds

^2 ^concurrent *Staphylococcus aureus *bloodstream infection

Table 4 shows the comparison of demographic data and clinical characteristics between patients who survived vs. those who died. Univariate analysis revealed that none of these variables was associated with mortality.

**Table 4:** Comparison of demographic data and clinical characteristics between patients who survived vs. those who died

Factors	Survive (N = 70)	Death (N = 12)	p-value
Male (N, %)	33 (47.1)	9 (75.0)	0.074
Age (years) (median, IQR)	50 (28, 63)	59 (48.5, 64.5)	0.115
Underlying diseases (N, %)			
Diabetes	16 (22.9)	1 (8.3)	0.275
Chronic renal diseases	9 (12.9)	1 (8.3)	0.661
Cirrhosis	1 (1.4)	1 (8.3)	0.206
Malignancy	6 (8.6)	2 (16.7)	0.791
Signs/symptoms (N, %)			
Fever	54 (77.1)	8 (66.7)	0.438
Headache	5 (7.1)	3 (25.0)	0.071
Alteration of consciousness	6 (8.6)	1 (8.3)	0.978
Site of infection (N, %)			
Bloodstream	24 (34.3)	5 (41.7)	0.621
Meningitis	5 (7.1)	3 (25.0)	0.054
Laboratory findings (mean ± SD or median, IQR)			
Hemoglobin (g/dL)	12.1 ± 2.1	12.6 ± 3.4	0.486
White blood cell count (cells/mm^3^)	12,700 (9800, 17100)	10,620 (7850, 15740)	0.213
Serum creatinine (mg/dL)	1.1 (0.9, 1.7)	1.6 (1.0, 2.8)	0.073

## Discussion

*Streptococcus agalactiae *is generally known to cause invasive infection in pregnant women and neonates since it commonly colonizes the vaginal and gastrointestinal tracts of healthy women[2]. However, infection in non-pregnant adults has been increasingly reported worldwide[5-8,10]. The incidence of invasive group B streptococcal diseases in the United States significantly increased from 3.4/100 000 population in 1999 to 5.0/100 000 population in 2005[10]. The same trend was observed in Asia[6,8]. In our study, although the number of cases increased significantly from 2006 to 2007 (p = 0.017, data not shown), the difference was not observed from 2007 to 2009. However, we at least demonstrated that there were 5 times more cases within 4 years given the similar diagnostic method and data collection. This study also supports the evidence of increasing number of patients who were not pregnant. In addition, infections in non-pregnant adults occurred in similar proportion in male and female that corresponded to the previous studies [3,6,8,9]

Other reports have not noted seasonal variation. However, our study revealed that the number was higher in September. We may need to further observe this phenomenon and explore the explanation for this finding.

Sixty percent of infected patients had underlying medical conditions including diabetes, chronic renal disease, and malignancy. Diabetes was the most common underlying medical disease in previous reports [2,7,10] which might be explained by the defect of bacterial engulfment by the neutrophils[19].

Isolation of *S. agalactiae *from urine were mostly from colonization; in our study, only 6.3% (5/79) of patients with positive urine culture were determined as true infection. This finding was similar to the report from the United States that specimens from urine and vaginal secretions were more likely to be colonization than infection[4].

The most common site of *S. agalactiae *infection was bloodstream infection which was also similar to other reports [2,3,6,7,10]. However, a study from Greece found that the most common site of infection was urinary tract[5]. In that study, the authors stated that there were two times more females than males, and it might be possible that faecal contamination during urine collection might have occurred[5]. Although echocardiogram was performed in all cases of bacteremia, no endocarditis was found in our study.

Clinical data comparing to other studies is shown in Table 5. Interestingly, in our study, meningitis was found in higher proportion than in other reports[3,6-8,10,11]. Seventy-five percent (6/8) of our patients with meningitis had concurrent bloodstream infection. The variation in infection sites among various reports might be attributed to the serotype of *S. agalactiae. S. agalactiae *is classified into serotype Ia/c, Ia/b, II, III, IV, V, VI, VII, and VIII[20]. Bolanos M, et al found that meningitis was correlated with infection with serotype Ia, whereas bloodstream infection, skin and soft tissue infections, urinary tract infection, and respiratory tract infections were correlated with infection with serotype III, V, III and V, and IV, respectively[21]. In addition, serotypes vary by geographic regions, e.g., serotype Ia, Ib, II, III, and V were accounted for 88% of adult cases in the United States and the most common serotype was serotype V[10]. In Japan, serotype Ia-V accounted for 69% of adult cases, and the most common serotype was serotype III[8]. This report also showed the virulence of serotype VI, VII, and VIII, which were not common in the United States[8,10]. However, serotyping was not performed in our study.

**Table 5:** Characteristics of *Streptococcus agalactiae *infections from various studies

Characteristics	Present study N = 82 2006-2009	Greece^5^ N = 26 1995-1999	Taiwan^7^ N = 94 2001-2003	Japan^8^ N = 52 1998-2007	Spain^9^ N = 51 1985-1994	Thailand^11^ N = 78 1997-2001
**1. Demographic data**						
Study population	Adults	Non-pregnant adults	Non-pregnant adults	Non-pregnant adults	Non-pregnant adults	Non-pregnant adults
Age (years) (mean, range)	48.5 (17-83)	57.7 (18-84)	64.7 (22-89)	62 (29-90)	63.3 (21-88)	55 (16-83)
Male (N, %)	42 (51.2)	-	46 (48.9)	27 (52.9)	27(53)	34 (43.6)
**2. Underlying diseases (N, %) **						
Diabetes	17 (20.7)	7 (20.6)	40 (42.6)	25 (48)	14 (27.5)	28 (36)
Malignancy	8 (9.8)	8 (23.5)	41 (43.6)	12 (23)	17 (33.3)	20 (26)
Cirrhosis	2 (2.1)	-	15 (16)	6 (11.5)	18 (35.3)	7 (9)
Chronic renal diseases	10 (12.2)	1 (2.9)	-	9 (17.3)	8 (17.7)	4 (5)
**3. Site of infection (N, %)**						
Bloodstream	29 (35.4)	2 (7.7)	32 (34)	12 (23)	20 (39.2)	24 (30.8)
Skin and subcutaneous	19 (23.2)	1 (3.8)	30 (31.9)	26 (50)	8 (15.7)	19 (24.4)
Lung	0 (0)	2 (7.7)	9 (9.6)	-	5 (9.8)	7 (9.0)
Trachea	1 (1.2)	-	-	-	-	-
Peritoneal cavity	1 (1.2)	1 (3.8)	7 (7.4)	1 (1.9)	5 (9.8)	6 (7.7)
Joint	8 (9.8)	-	3 (3.2)	-	-	19 (24.4)
Meninges	8 (9.8)	-	2 (2.1)	2 (3.8)	-	2 (2.6)
Urinary tract	5 (6.1)	13 (50)	3 (3.2)	3 (5.8)	6 (11.8)	1 (1.3)
Endocardium	0	-	4 (4.3)	1 (1.9)	1 (2)	7 (9.0)
**4. Mortality (N, %)**	12 (14.6)	1 (3.9)	19 (20.2)	8 (15.4)	17(33.3)	19 (24.3)

The mortality rate in our study was 15% while those in other reports varied from 3 to over 30%[5,7-9,11]. Other reports found that mortality was associated with bloodstream infection, hypotension, thrombocytopenia, other concurrent bacterial infections, infection with serotype Ia, and old age[2,3,7-10]. Although we found that 83.3% (10/12) of death occurred in patients who had severe diseases, i.e., bloodstream infection and/or meningitis, we failed to demonstrate this association in the univariate analysis. In addition, we could not identify other factors associated with mortality, which is probably due to the small sample size.

All isolates in our study were susceptible to penicillin, ampicillin, and vancomycin, which was similar to other reports[5,22,23]. In general, group B streptococci remain uniformly susceptible to penicillins and cephalosporins in vitro, and penicillin G is still the drug of choice once the diagnosis is established[22]. However, ceftriaxone was frequently used in our study due to the fact that it could be administered once daily. Vancomycin, an alternative choice in patients who are allergic to β-lactams, was less frequently prescribed in our study.

## Conclusions

*S. agalactiae *infection is a growing problem in non-pregnant patients, particularly in patients who had underlying medical conditions. Physicians should consider infection with *S. agalactiae *in the differential diagnosis of patients with meningitis or septicemia. It is noticeable from our study that no pregnant women with *S. agalactiae *chorioamnionitis had life-threatening conditions including bloodstream infection and meningitis, whereas non-pregnant patients had more serious infections; this might be explained by the fact that pregnant women are younger and have less comorbidities.

## Competing interests

The authors declare that they have no competing interests.

## Authors' contributions

RC participated in the design of the study, performed the statistical analysis, and drafted the manuscript. WJ participated in data collection and performed the statistical analysis. MB participated in data collection. NN participated in data collection. KS and TS revised manuscript critically for important intellectual content. All authors read and approved the final manuscript.

## Pre-publication history

The pre-publication history for this paper can be accessed here:

http://www.biomedcentral.com/1471-2334/11/149/prepub
